# Improvement of recalcitrant Hailey–Hailey disease (*ATP2C1*-nonsyndromic epidermal differentiation disorder) with topical ruxolitinib 1.5% cream

**DOI:** 10.1093/skinhd/vzag002

**Published:** 2026-03-13

**Authors:** Alyssa Jobe, Ashley Ng, Shanelle Briggs, Philip C Berce, Maia K Keller, Karolyn A Wanat

**Affiliations:** Department of Dermatology, Medical College of Wisconsin, Wauwatosa, WI, USA; Department of Dermatology, Medical College of Wisconsin, Wauwatosa, WI, USA; Department of Dermatology, Medical College of Wisconsin, Wauwatosa, WI, USA; Department of Dermatology, Medical College of Wisconsin, Wauwatosa, WI, USA; Department of Dermatology, Medical College of Wisconsin, Wauwatosa, WI, USA; Department of Dermatology, Medical College of Wisconsin, Wauwatosa, WI, USA

## Abstract

*ATP2C1*-nonsyndromic epidermal differentiation disorder, also known as familial benign chronic pemphigus or Hailey–Hailey disease, is a rare autosomal dominant acantholytic disorder characterized by relapsing and remitting painful vesicles and erosions in flexural areas. At present, there is no effective cure. Current treatments focus on symptomatic management and improving quality of life. We report a case in which a patient experienced notable clinical improvement with the use of topical ruxolitinib 1.5% cream.

## Teaser

This report highlights the use of topical ruxolitinib 1.5% cream monotherapy in the treatment of relapsing and remitting Hailey–Hailey disease. There is currently no cure for Hailey–Hailey disease, and treatment revolves around symptomatic management. Our findings include rapid pain relief and sustained periods of remission offering a novel therapeutic avenue.

What is already known about this topic?

- There is no current cure for *ATP2C1*-nonsyndromic epidermal differentiation disorder (also known as Hailey–Hailey disease; HHD); management is largely symptomatic.

What does this study add?

- We present a patient with multifocal, recalcitrant HHD treated with ruxolitinib monotherapy with rapid resolution of flares.

## Case report

**Figure 1:**
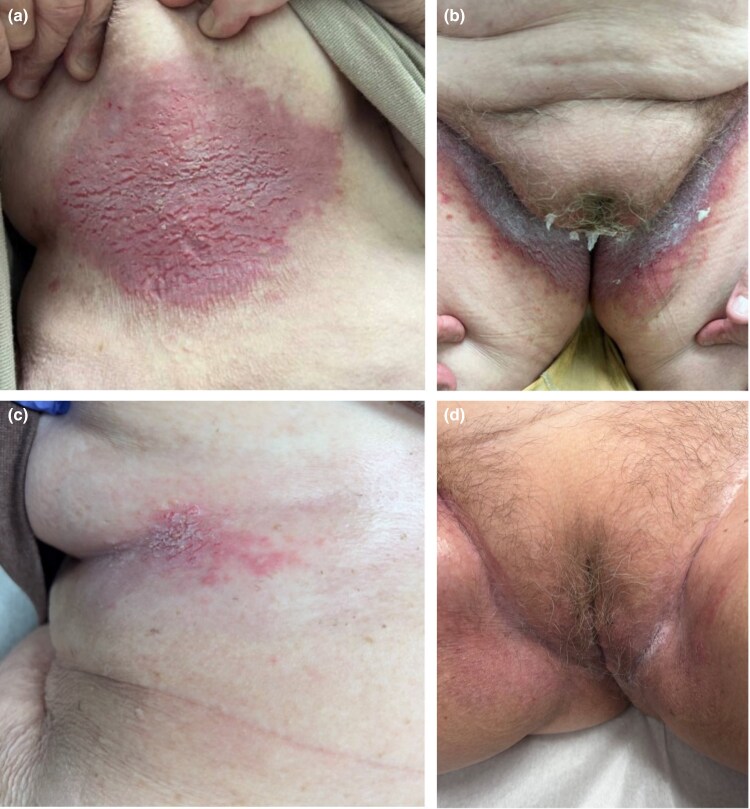
Clinical images demonstrating clinical findings (a, b) before and (c, d) after 3 months of treatment with twice-daily topical ruxolitinib 1.5% cream. (a) Inframammary fold pre treatment. (b) Inguinal fold pre treatment. (c) Inframammary fold post treatment. (d) Inguinal fold post-treatment.

A 77-year-old woman with a diagnosis of *ATP2C1* nonsyndromic epidermal differentiation disorder (*ATP2C1*-nEDD), supported by histopathology and positive family history of the disorder in her daughter, presented with a long-standing history of severe, recurrent, fluctuating, painful lesions involving the inframammary and inguinal folds. Examination was notable for sharply demarcated, macerated and fissured, grey–pink plaques in the inframammary and inguinal creases (Figure 1a, b). Her flares were recalcitrant to multiple treatments, including topical vitamin D analogues, topical and oral antibiotics, topical corticosteroids, topical calcineurin inhibitors, oral retinoids, topical antifungals, oral low-dose naltrexone, topical cinacalcet ointment, elemental magnesium, adalimumab and apremilast. Her pain was persistent and debilitating, requiring referral to a pain management specialist.

After discontinuing previous agents, the patient was prescribed topical ruxolitinib 1.5% cream with which she reported rapid pain relief with initial application, and resolution of her *ATP2C1*-nEDD after 3 days of twice-daily application (Figure 1c, d). Due to the high out-of-pocket cost, the patient used ruxolitinib cream sparingly outside of acute flares. However, periods of remission lasted for several weeks, and subsequent flares were milder and responded more quickly compared with previous treatments. The patient tolerated treatment well without irritation or other reported side effects.

## Discussion

*ATP2C1*-nEDD is a rare autosomal dominant disorder caused by mutations in *ATP2C1* leading to defective calcium regulation in keratinocytes and loss of cell-to-cell adhesion.^1^ While various topical and systemic treatments have been utilized off-label in the treatment of *ATP2C1*-nEDD, few have demonstrated lasting or consistent efficacy in symptom management. Furthermore, individuals may respond to each treatment with varying degrees of efficacy.

To our knowledge, there are only two other reports of recalcitrant *ATP2C1*-nEDD responding to topical ruxolitinib 1.5% cream. In one case, a patient with long-standing refractory disease had partial improvement with dupilumab and required concomitant ruxolitinib 1.5% cream for refractory lesions affecting the inguinal folds.^2^ In another case, a patient with severe anogenital *ATP2C1*-nEDD responded to ruxolitinib 1.5% cream alone.^3^ Similar to our patient, both of the other patients demonstrated rapid resolution of skin lesions with twice-daily application of ruxolitinib cream and good tolerability of the medication. Notably, our case illustrates that topical ruxolitinib monotherapy may be a reliable treatment, even in patients with multifocal disease.

The mechanism by which topical ruxolitinib works in the treatment of *ATP2C1*-nEDD is not well understood. *In vitro* research has demonstrated that the addition of anti-inflammatory antibodies, specifically anti-interleukin (IL)-6 and anti-IL-8, restored proper *ATP2C1* expression in human keratinocytes.^4^ The authors speculate that the anti-inflammatory properties of ruxolitinib, via inhibition of the Janus kinase (JAK) 1 and JAK2 pathways, may work through a similar mechanism thereby suppressing pro inflammatory cytokines and enhancing *ATP2C1* expression. Given the recent evidence supporting dupilumab use in *ATP2C1*-nEDD, it is possible that ruxolitinib may act by inhibiting IL-4 and IL-13, which utilize the JAK1 and JAK2 pathways and are thought to be important in regulating intracellular calcium release.^5^ For patients requiring additional therapy, abrocitinib has also demonstrated a robust and sustained response, offering a systemic alternative for patients who are unable to apply or tolerate topical ruxolitinib.^6^

This case adds to the limited literature regarding the promise of JAK inhibition in the treatment of *ATP2C1*-nEDD, through the successful use of topical ruxolitinib cream in a patient with multifocal, recalcitrant and debilitating *ATP2C1*-nEDD.

### Author contributions

Alyssa Jobe (Writing—original draft [lead]), Ashley Ng (Writing—original draft [equal], Writing—review & editing [equal]), Shanelle Briggs (Writing—review & editing [equal]), Philip C Berce (Writing—review & editing [equal]), Maia K Keller (Writing—review & editing [equal]), and Karolyn A Wanat (Writing—review & editing [equal])

## Data Availability

The data underlying this article will be shared on reasonable request to the corresponding author.

## References

[1] Hu Z, Bonifas JM, Beech J et al Mutations in *ATP2C1*, encoding a calcium pump, cause Hailey-Hailey disease. Nat Genet 2000; 24:61–5. 10615129 10.1038/71701

[2] Khang J, Yardman-Frank JM, Chen L-C, Chung HJ. Recalcitrant Hailey-Hailey disease successfully treated with topical ruxolitinib cream and dupilumab. JAAD Case Rep 2023; 42:56–8. 38058412 10.1016/j.jdcr.2023.10.004 PMC10696304

[3] Ly S, Beylot-Barry M, Seneschal J. Refractory anogenital Hailey-Hailey disease successfully treated with topical ruxolitinib. J Eur Acad Dermatol Venereol 2025; 39:e786–7. 40028665 10.1111/jdv.20620

[4] Mayuzumi N, Ikeda S, Kawada H, Ogawa H. Effects of drugs and anticytokine antibodies on expression of *ATP2A2* and *ATP2C1* in cultured normal human keratinocytes. Br J Dermatol 2005; 152:920–4. 15888147 10.1111/j.1365-2133.2005.06394.x

[5] Alzahrani N, Grossman-Kranseler J, Swali R et al Hailey–Hailey disease treated with dupilumab: a case series. Br J Dermatol 2021; 185:680–2. 33971025 10.1111/bjd.20475

[6] Li Y, Jiang Y, Sun J. Improvement of Hailey-Hailey disease with abrocitinib. Clin Exp Dermatol 2023; 48:532–3. 36723952 10.1093/ced/llad023

